# Instant Stent-Accentuated Three-Dimensional Optical Coherence Tomography Guided Selection of Proper Distal Cell for Side Branch Dilatation in Bifurcation Stenting

**DOI:** 10.1155/2015/915084

**Published:** 2015-05-31

**Authors:** Fumiaki Nakao

**Affiliations:** Department of Cardiology, Yamaguchi Grand Medical Center, 77 Ohsaki, Hofu, Yamaguchi 747-8511, Japan

## Abstract

In the bifurcation stenting, the distal rewiring for the side branch postdilatation confirmed by two-dimensional modalities may not lead to favorable results in some cases. If there are two distal cells divided by the link bridging from the carina, the rewiring through the larger distal cell may be recommended for the side branch postdilatation. Detailed confirmation of the rewired cell by the intraprocedural instant stent-accentuated three-dimensional optical coherence tomography is important.

## 1. Introduction

In the bifurcation stenting, the incomplete stent apposition (ISA) is a factor of poor prognosis and the proximal rewiring for the side branch (SB) postdilatation is a cause of the ISA [1]. Okamura et al. reported using the stent-enhanced three-dimensional optical coherence tomography (3D-OCT) in which jailed patterns of the SB ostium were divided into two types according to the presence of the link bridging from the carina (the free carina type, the connection to carina type), the distal rewiring was defined as the rewiring through the area which was enclosed by the carina and the stent strut having at least one distal top of the stent hoop located in front of the SB ostium, and both the free carina type and the distal rewiring lead to reducing the ISA after the SB postdilatation [2]. Detailed confirmation may be required to obtain a good result in the connection to carina type, because of the presence of two distal cells.

The instant stent-accentuated 3D-OCT (iSA3D-OCT) was developed to confirm the relation between stent struts and the jailed SB and the rewired cell in the bifurcation stenting [3]. It takes about 30 sec to automatically reconstruct the longitudinal cutaway view of the iSA3D-OCT from the original two-dimensional (2D) OCT by the offline computer with freeware ImageJ 1.47v (National Institutes of Health) and self-made macroprograms. The processing time and the quality of images of the iSA3D-OCT are acceptable for the clinical use [3, 4].

## 2. Case Report

**Figure 1:**
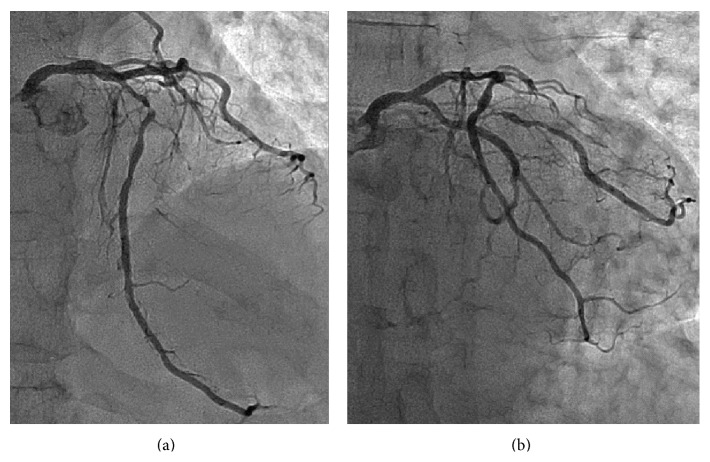
Baseline coronary angiography. (a) Cranial view and (b) caudal view.

**Figure 2:**
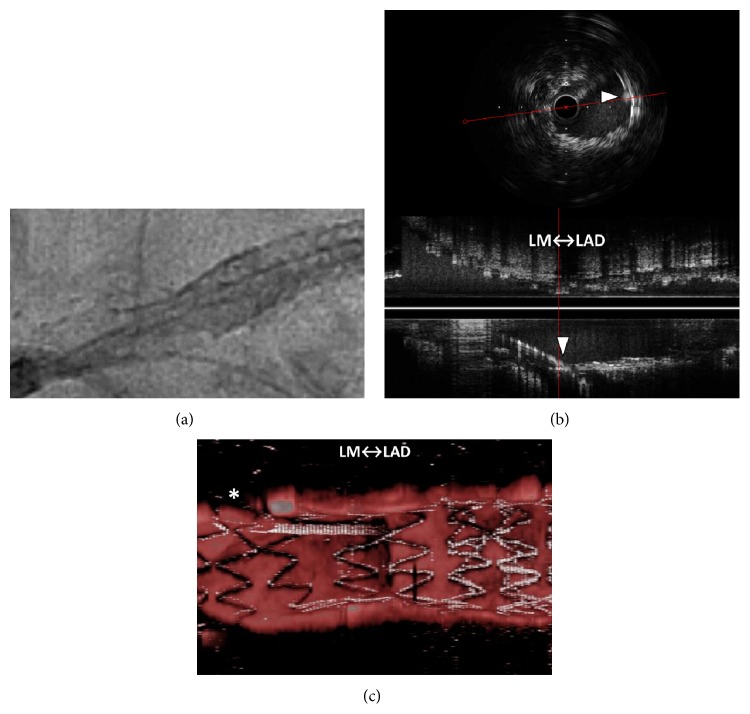
First rewiring. (a) X-ray fluorography, (b) intravascular ultrasonography showing the distal rewiring (arrowheads), and (c) intraprocedural instant stent-accentuated three-dimensional optical coherence tomography showing the rewiring through the smaller distal cell. LM: left main coronary artery, LAD: left anterior descending artery, and *∗*: guide wire shadow artifact.

**Figure 3:**
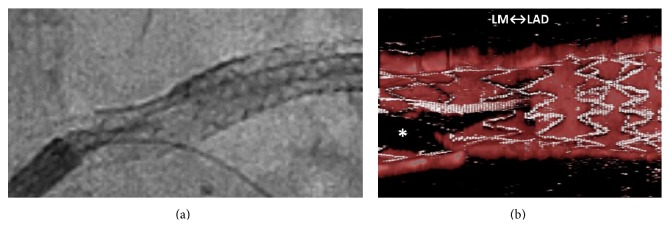
Third rewiring. (a) X-ray fluorography, (b) intraprocedural instant stent-accentuated three-dimensional optical coherence tomography showing the rewiring through the larger distal cell. LM: left main coronary artery, LAD: left anterior descending artery, and *∗*: guide wire shadow artifact.

**Figure 4:**
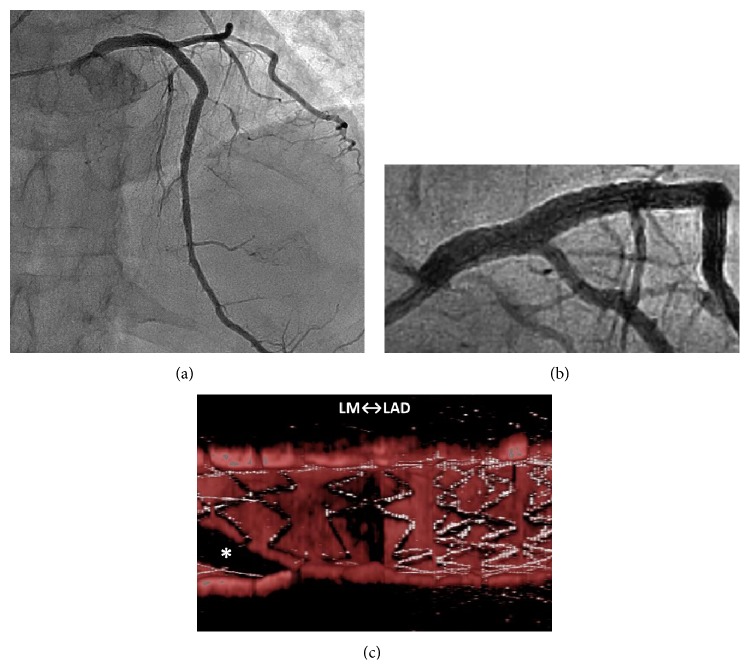
Final results. Cranial view (a) and caudal view (b) of final coronary angiography. (c) Final instant stent-accentuated three-dimensional optical coherence tomography showing minimized floating struts. LM: left main coronary artery, LAD: left anterior descending artery, and *∗*: guide wire shadow artifact.

A 69-year-old man underwent percutaneous coronary intervention (PCI) for stenoses of the left anterior descending artery (LAD) (Figure 1). The ostial-mid LAD was stented with two platinum-chromium everolimus-eluting stents (Promus Premier, Boston Scientific Co.). Additional bail-out stenting was required for the proximal stent-edge dissection. The left main coronary artery-proximal LAD was stented with a 3.5 × 14 mm two-link ten-crown biolimus-eluting stent (Japanese design of Nobori, Terumo). After the guide wire was rewired to the left circumflex artery (LCx) (Figure 2(a)), intravascular ultrasonography (IVUS, OptiCross, Boston Scientific Co.) showed the distal rewiring (Figure 2(b) arrowheads). Intraprocedural iSA3D-OCT made from 2D-OCT (Dragonfly JP and ILUMIEN Optis, St. Jude Medical) showed two distal cells (the connection to carina type) and the rewiring through the smaller distal cell (Figure 2(c)). The larger distal cell located on counterclockwise direction for the smaller distal cell. The second rewiring was the same as the first rewiring. At the third rewiring (Figure 3(a)), the guide wire successfully passed through the larger distal cell (Figure 3(b)). After the kissing balloon postdilatation was performed by simultaneously inflating 3.5 mm and 2.0 mm balloons, the final coronary angiography showed a good result (Figures 4(a) and 4(b)), and the final iSA3D-OCT showed minimized floating struts (Figure 4(c)).

## 3. Discussion

Although generally the distal rewiring on the SB ostium for the SB postdilatation has been recommended [5], the distal rewiring confirmed by 2D modalities may be insufficient to reduce the ISA. It is difficult to control the position of the link. In the connection to carina type that is present in 46% of the bifurcation stenting [2], there are two distal cells divided by the link bridging from the carina, and therefore further care may be required in addition to the distal rewiring. The rewiring through the larger distal cell is further advantageous than the smaller distal cell, in the point of the reduction of the ISA and the crossability to the SB lesion.

Another problem about the distal rewiring confirmed by 2D modalities is the rewiring through the far distal cell [4]. The far distal cell is defined as the area which is enclosed by the carina and the stent strut having no distal tops of the stent hoop located in front of the SB ostium. The dilatation of the far distal cell may lead to floating struts on the SB ostium and struts covering sparsely the distal main vessel. When the postdilatation of the SB ostium was required, the rewiring through the large center cell may be better than the far distal cell, even if the metallic carina is made.

In this case, at the first rewiring, the IVUS showed the distal rewiring. On the other hand, the intraprocedural iSA3D-OCT showed two distal cells and the rewiring through the smaller distal cell. The rewiring through the larger distal cell seemed to be advantageous for the postdilatation of the SB ostium. At the third rewiring, the guide wire was passed through the larger distal cell. In another day, there were no troubles in PCI for the LCx.

The distal rewiring confirmed by 2D modalities may not lead to favorable results in some cases. If there are two distal cells divided by the link bridging from the carina, the rewiring through the larger distal cell may be recommended for the SB postdilatation. Detailed confirmation of the rewired cell by intraprocedural iSA3D-OCT is important to find such cases.
